# Cambodia’s health professionals and the ASEAN Mutual Recognition Arrangements: registration, education and mobility

**DOI:** 10.1186/s12960-019-0349-5

**Published:** 2019-02-26

**Authors:** Kristy Meng-Hsi Law, Vannarath Te, Peter S. Hill

**Affiliations:** 1 School of Public Health, The University of Queensland, Herston, Brisbane, Australia; 2 Ministry of Health, National Institute of Public Health, Phnom Penh, Cambodia

**Keywords:** Cambodia, ASEAN, Mutual Recognition Arrangements, Human resources, Doctors, Dentists, Nurses, Midwifery, Registration, Mobility

## Abstract

**Background:**

From 2006, the Association of South East Asian Nations (ASEAN) has been developing Mutual Recognition Arrangements (MRAs) across key professions, including medicine, dentistry and nursing, that would facilitate the development of an ASEAN Economic Community, with shared regional standards and easier mobility of the workforce. This paper examines the interface between those agreements and the registration, professional education and mobility of health personnel in Cambodia.

**Methods:**

This qualitative health policy analysis combined documentary and policy review with key informant interviews with 16 representatives of agencies relevant to the development and implementation of the MRAs in health. Thematic analysis identified three themes: registration, education and mobility.

**Results:**

Cambodia is an active participant in the ASEAN MRA processes for doctors, dentists and nurses reporting progress annually. Education of health professionals has been increasingly formalised in the past 25 years, with nursing moving towards a 4-year bachelor degree. The private university sector has substantially increased, with English increasingly used as a language of instruction. Recent legislation provides for enforcement through fines and/or imprisonment to ensure all practising health professionals hold initial registration as a health professional and a renewable licence to practise as a health practitioner. Continuing Professional Development is a mandatory requirement for licence renewal. This is consistent with the MRA guidelines, though the capacity for enforcement appears limited. The Medical Council of Cambodia (MCC), and more recently, the Dental and Nursing Councils, have introduced continuing professional development initiatives, using them strategically as a positive reinforcer of registration. Midwifery education and registration in Cambodia does not conform with ASEAN guidelines. In education, course durations in medicine and dentistry are longer than regional counterparts, though anxiety around maintaining clinical standards has resulted in the introduction of a National Exit Examination and reluctance to abbreviate courses. The introduction of reforms appears to reference regional standards, though parity is still some way off. Mobility at present is infrequent and more likely to result from informal mechanisms than through the MRA mechanisms.

**Conclusion:**

The Royal Government of Cambodia is committed to the ASEAN MRA process. Developments in registration appear to use regional standards as benchmarks, as do reforms in the education of health professionals, though domestic factors appear to more directly impact on developments. Informal mechanisms facilitate the limited mobility currently occurring, with little formal application of the MRA provisions evident at this point.

## Background

In 2015, the Association of South East Asian Nations (ASEAN) announced the establishment of the ASEAN Economic Community (AEC) [1], loosely modelled after the European Economic Community. The AEC was envisaged as a single market and production base, developing equitably to compete economically in the global economy. Equitable development and the free flow of trade in services and skilled labour were identified as critical to achieving this [2]. Given the extraordinary diversity across ASEAN—with economies ranging from Singapore to Myanmar—Mutual Recognition Arrangements (MRAs) were established in seven key occupations where facilitated mobility, information exchange on standards and qualifications, promotion of best practice and opportunities for capacity building and professional education were seen as prerequisites to regional development. Health was an early focus, with MRAs in nursing (2006) and medicine and dentistry (2009). However, development of the MRAs in health has been cautious: while ASEAN Joint Coordinating Committees (AJCC) have been established for each health discipline, governance remains at the member state level and regional mechanisms such as a professional registry or secretariat have not been created as with other professions [3], resulting in inconsistent implementation of the MRA process [4]. A recent scoping review of the MRAs in health provides broad insight into their implementation, though there is limited documentation of country experience available [5].

Cambodia—the last of the current 10 member states to join in 1999—illustrates both the advantages and challenges of MRA implementation. Despite its recent elevation to low-middle income status, it is clustered with Lao PDR, Myanmar and Vietnam, distinct from the six more advanced economies. With the health workforce decimated during the 1975–1979 Khmer Rouge period, and the decade of international sanctions that followed, the impact on Cambodia’s health professions is still evident [6] and shapes its response to the MRA process. The early initiatives to repopulate the health workforce saw accelerated medical education, with medical assistants and experienced nurses “upgraded”, effectively removing the cohort that could have provided mature nursing leadership [7]. The accelerated education reiterated the hospital orientation and theoretical emphasis of the French curricula of the 1970s, but initially forgoing the thesis. In the re-establishment of educational institutions, French was the preferred language of instruction complemented with Khmer, with French development assistance concentrated in the (then) Université des Sciences de la Santé (now University of Health Sciences) and the Hôpital Calmette, with Dentistry and Pharmacy faculties within the Université. Nursing and midwifery were taught through the École Centrale des Cadres Sanitaires and two regional schools, in courses of between 1 and 3 years. Non-government organisations trained a range of health professionals in border camps, and the integration of demobilised military health staff into the health system left the country with a bloated workforce of uncertain quality.

But the past 25 years have seen rapid demographic and economic transformation in the wake of political stability and international solidarity [8], with Cambodia now emerging as a middle-income economy. The health sector reforms of the late 1990s have set up the infrastructure for universal health coverage; over the life of the Millennium Development Goals, health status has measurably improved. Early experience with contracting out of District Health Services has been extended with direct government funding [9]. Health Equity Funds provide a level of protection to the marginalised. Curricula in medicine, dentistry and nursing have been revised, with nursing now commencing a 4-year Bachelor’s degree programme. Private universities teaching medicine, dentistry and nursing have recently evolved, with English assuming dominance with Khmer as the language of instruction, consistent with Cambodia’s ASEAN neighbours. The timing would appear to be optimal for Cambodia to leverage the MRAs to ensure the quality and regional recognition of its health workforce, to share resources and best practice experience and to explore the potential for capacity building and professional development. This study seeks to provide an overview of the Cambodian experience in its engagement with the ASEAN MRAs in medicine, dentistry and nursing and to analyse its influence on Cambodian registration, education and mobility of health personnel.

## Methods

This research case study used qualitative research methods combining literature review with key informant interviews to examine the interface between the ASEAN MRAs and the registration of health professionals (doctors, dentists and nurses) in Cambodia, their education and mobility within the region.

A literature review identified 11 relevant peer-reviewed articles from six electronic databases: PubMed, ProQuest, EBSCO, CINAHL, Scopus and Web of Science, published in English since 2007. The search followed the RAMESES guidelines for meta-narrative review [10], using keywords relating to the registration, education and mobility of medical, dental and nursing practitioners in Cambodia. This was supplemented with a web-based search of the websites of ASEAN (specifically those related to the Joint Coordinating Committees), Cambodian Ministry of Health (MoH) and professional councils and international agencies directly involved in human resources for health (Table 1). Key informant interviews were undertaken over a 2-week period in April 2017 with 16 participants identified as being directly involved in the education or registration of doctors, dentists and nurses. The characteristics of the informants ensured a rich mix of experience: nine Khmer informants, seven expatriates; eight working in Cambodian institutions (both public and private), eight in international agencies, including one hospital; ten males, six females; and approximately equal distributions in terms of professional backgrounds (five each from medicine and nursing, four from dentistry and two others). Informants’ interview transcripts were de-identified and allocated codes, used in this paper to track quotations (#001--#16). Further identification—by discipline, employer, gender or ethnic group—risks confidentiality of informants.

**Table 1:** Organisations engaged in health workforce education, registration and support

Ministry of Education, Youth and Sports	Ministry of Health	Cambodian Health Profession Councils	Health Professional Associations	International Agencies
Accreditation Committee of Cambodia (ACC)		Medical Council of Cambodia (MCC) (National)	Medical Association of Cambodia	World Health Organization
University of Health Sciences	Provincial Health Departments	Dental Council of Cambodia (DCC) (National)	Pharmacist Association of Cambodia	United Nations Population Fund
Technical School of Medical Care (TSMC)	National Teaching Hospitals	Cambodian Midwives Council (CMC) (National)	Dental Association of Cambodia	World Bank
Private Universities and training agencies	National Institute of Public Health	Cambodian Council of Nurses (CCN) (National)	Cambodian Nurses Association	Deutsche Gesellschaft für Internationale Zusammenarbeit
		Pharmacy Council of Cambodia (PCC) (National)	Cambodian Midwives Association	Japan International Cooperation Agency
		Regional Councils	Nursing and Midwifery Education Society of Cambodia	Korea International Cooperation Agency
		Provincial Councils		United States Agency for International Development

**Fig. 1:**
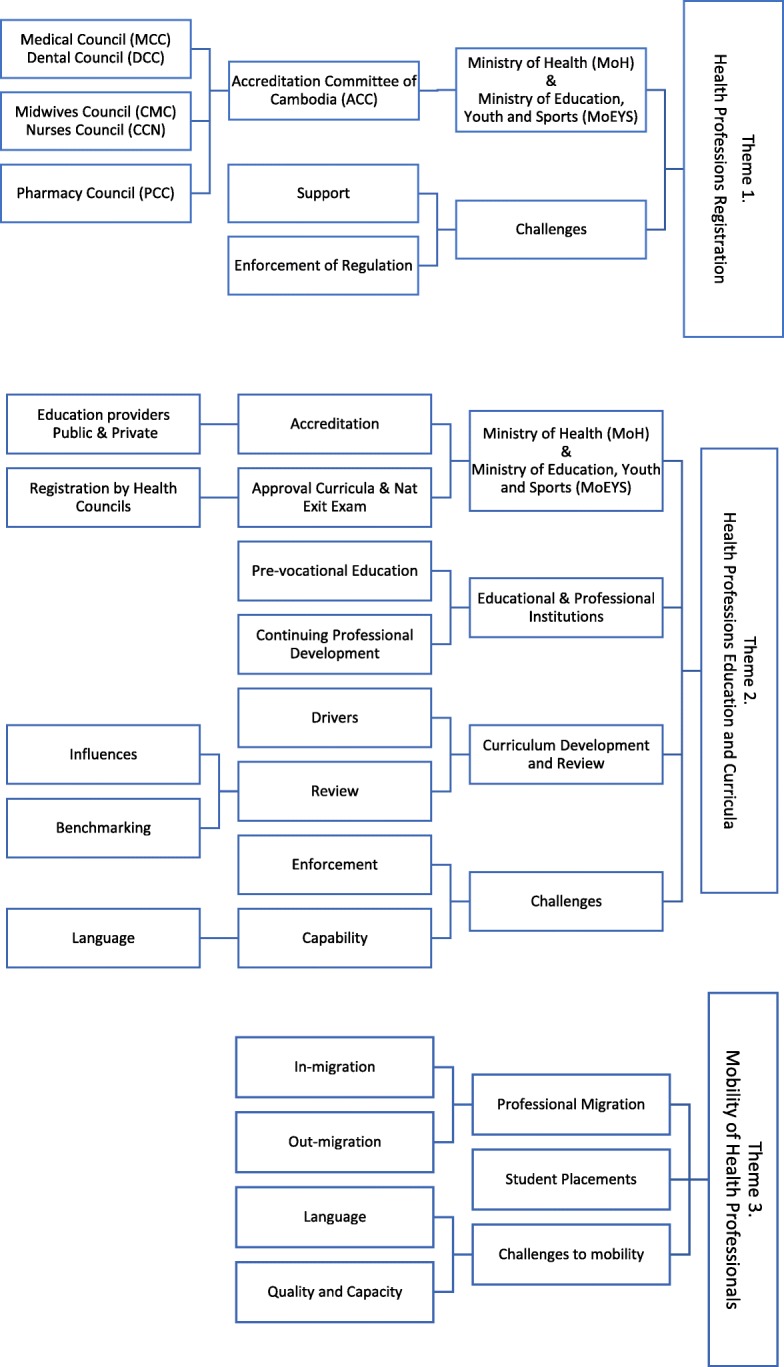
Thematic map for analysis

Participants were contacted directly by phone or email and provided with an information sheet and consent form in English and Khmer, with written consent prior to interview. Interviews were conducted face to face at a location nominated by the interviewee, usually their place of work, but in three cases, in cafes, one interview was conducted by skype. Participation was voluntary, and participants were advised that they could withdraw at any point and that any information provided would be de-identified prior to publication. The interviews were conducted in English or Khmer and recorded, translated and transcribed for analysis. Three a priori themes were identified by the authors from the research proposal: health professionals’ registration, health professionals’ education and curricula and mobility of health professionals (Fig. 1). Given the limited number of interviews, KL coded the papers manually using a combination of a priori sub-themes from the literature review, and emergent themes arising from the analysis of the interviews. Findings from the narrative literature review and the interviews have been integrated in the presentation of the results [11].

## Results

In 2016, the Cambodian MoH released its third *Health Workforce Development Plan* [12]. It dealt with the key issues addressed by the MRA—concerns with education standards, the quality of education and appropriate clinical exposure and the challenges with regulating a growing and diverse health workforce—but without specific reference to ASEAN processes. But Cambodia’s ASEAN neighbours remain an immediate frame of reference—with Thailand and Vietnam (#005) and the Philippines (#012) providing relevant benchmarks:

Most of them, when they start to develop their guidelines will… look across – particularly across ASEAN in the first instance and across the region. (#002)

Despite ASEAN’s colonial, cultural, ethnic and economic diversity, the two decades since Cambodia’s admission in 1999 have been characterised by increasing convergence: transitioning economically from post-conflict donor-dependence to middle-income status, engaging increasingly with regional development partners (especially Japan, Korea, China) and reorienting from French towards ASEAN’s preference for English in education, diplomacy and commerce. In this, the Cambodian MoH has been a lead ministry in terms of internal reform, and potentially in international developments.

### Registration in Cambodia and the MRAs

With the universities—including the flagship University of Health Sciences—now accountable to the Ministry of Education, Youth and Sport (MoEYS), and the introduction of the new law on Regulation of Health Practitioners in late 2016, responsibility for registration is a shared responsibility between MoEYS and MoH. The five professional councils set the Scope of Practice for their profession and propose competencies for graduates: Medical Council of Cambodia (MCC), Dental Council of Cambodia (DCC), Cambodian Midwives Council (CMC), Cambodian Council of Nurses (CCN) and Pharmacy Council of Cambodia (PCC) [13]. Maintaining independence is difficult, given the multiple roles played by some key actors across agencies:

…there’s been huge difficulty in separating the difference between a regulator and an association that advocates for the health profession… Nursing and medicine are the two that are struggling the most to separate council and professional association. (#002)

The MRAs currently include medicine, dentistry and nursing: there is no separate MRA for midwifery. Compliance with the MRAs in terms of registration is an acknowledged goal:

We’ve tried to align more with Asia than with the West for the reasons you’d expect, because it’s the region in which they’ll operate and work. So, registration, license to practice are a model for the region... (#002)

But while the values implicit in the MRAs are shared, the specific mechanisms of professional registration appear driven by internal histories and local commitment to improve standards, rather than a strategic use of the MRAs to drive reform from outside. The 2016 law on the Regulation of Health Practitioners makes registration compulsory for doctors, dentists and nurses, though registration of medical practitioners does not currently differentiate specialist practitioners. Awareness of the new requirement is high:

Recently we all hear about registration. Everybody had to register. Every profession, nurse, midwife, medical doctor, everybody had to be registered with their actual council. (#013)

Despite severe sanctions of up to 2 years imprisonment and US$2500 fines for failure to meet the requirements of registration [13, 14], enforcement mechanisms are inadequately resourced. Continuing Professional Development (CPD) is a requirement for ongoing licence renewal for all health professionals, but also is being used by both medicine and dentistry as an incentive for promoting registration to existing practitioners. The introduction of CPD is also consistent with MRA requirements.

Medicine used it as a mechanism to drive registration. Because people were saying what’s in it for me, why would I get registered, why would I want to be a member of a council? So, they have been going around the country running CPD sessions. Now that’s been quite successful. (#002)

The Dental Association has a meeting every year with overseas speakers, plus some local speakers, and these are inexpensive, with lots of sponsorship from companies that help support it, you see. And then the Dental Council has another conference later in the year, and there’s a lot of short courses in various areas, like implants or orthodontics. There’s a lot offered with visiting overseas speakers and so on. So for dentists, there’s lots of opportunities for continuing education. (#011)

The Nursing Council has been less successful in establishing CPD, given the lower incomes of their members, and limited donor or commercial support, but the recently created Nursing Midwifery Education Society of Cambodia, an active, voluntary initiative of 30 internationally trained graduate nurses, is offering biannual seminars:

This is the association that we provided. We would like to support our society—we promote nursing promotion. Our vision is to train nurses. Every year we have two seminars conducted by this group. (#006)

### Health professional education in Cambodia and the MRAs

Within the expanding education sector, the Accreditation Council of Cambodia (ACC), reporting to the MoEYS and MoH, accredits public and private education of health professionals, focusing on the foundation year programme and the quality of higher education institutions [15]. At review, its procedures were considered adequate, though clinical competence is not assessed in accreditation:

the documentation is very good, you know; it’s very similar, from what I’ve seen, to other accreditation processes overseas. (#011)

Education for doctors and dentists and degree courses for nurses are concentrated in the capital, with a strong hospital focus and limited clinical placements. To safeguard clinical standards, the MoH retains a direct interest. For medicine and dentistry, this has resulted in curricula longer than those of their ASEAN counterparts: “an eight year program for a medical doctor,” (#005; #008) when

many countries like even Malaysia, Singapore, and Australia and New Zealand, all of them, they have very short programs. (#004)

Concerns over clinical standards at registration have seen the introduction of national entry and exit examinations for medicine, dentistry and degree students in nursing, in addition to university certification. The National Exit Examination is managed by the National Examination Committee, chaired by the Council of Ministers with members from MoH, MoEYS, ACC and all public and private education institutions. It comprises both multiple choice and objective structured clinical examination (OSCE) and is required for registration. The heavy resource demands saw the OSCE suspended in 2017, amid concerns among regulators: “multiple choice questions cannot test the student competence—we need OSCEs.” (#008).

Despite requests for curriculum review that would shorten both medical and dentistry curricula, the MoH has remained conservative, concerned that clinical standards not be further risked, with review committees proceeding cautiously. A shared mandatory Foundation Year for all health professional students seeks to redress uneven secondary schooling and establish a common platform for the health workforce, but lengthens overall course length. Policy and review processes appear to be aware of MRA standards, but direct application has proved difficult to achieve with current resources:

We discuss the ASEAN full competency for a professional framework… We sit together, but before that they, every country select their own competency and then we… make the framework for ASEAN countries, for the 10 countries and we’re clear on that we have 5 domains… but each of the domains must be determined by the context of the country*.* (#012)

The core competencies we’ve developed are a little bit high and… still too low to compare with other countries. (#012)

Q. Was the MRA taken into consideration when designing your first draft?

A. Maybe no. I have no idea of these things. (#013)

Within the university sector, curriculum exploration is proceeding independently: the University of Health Sciences offers an international programme, with intensive courses in English and French and exposure to international academics [16]; the private dental schools are in dialogue with regional partners—though not through MRA mechanisms—seeking to secure MOH action to review their 7-year programme:

The review came about, actually, because we wrote a letter to the Ministry and we asked for a review of the curriculum, so they set up a technical working group and invited members from each university to be in that group and so it is under them; it is their group, and it’s their review. (#011)

Nursing faces particular issues in complying with the MRAs. With the additional mandatory Foundation Year, the 4-year degree course is effectively 3 years—a year less than the ASEAN 4-year degree standard. International cooperation is increasingly supporting nursing with strategies to bridge the MRA requirements, with the Japanese International Cooperation Agency (JICA) funding senior 30 nurses to complete their Bachelor’s degree in Thailand, providing a small but critical mass of experienced and ASEAN standard qualified nurses. The impact of this initiative has been reflected in increased activity through the Nursing Midwifery Education Society of Cambodia, but there is an emerging tension between mature diploma-trained nurses and the emerging cohort of degree qualified, but as yet less experienced graduates. The Korean International Cooperation Agency has supported bridging course to upgrade nurses, but is “only training the lecturers first”*,* (#006) as faculty staff are themselves mostly 3-year diploma-trained.

Although nursing education within the capital is delivered through the universities, “the regional training sectors work directly through to the ministry.” (#007). This ambiguous positioning of regional nursing education compounds the impact of the hierarchy of professions and institutions—with medicine dominating, followed by pharmacy and then dentistry, with nursing and midwifery education only recently being upgraded to university degree programmes.

Faced with the challenges of high maternal and neonatal mortality, Cambodia has launched a package of successful interventions [17], among them the introduction of the Associate Degree in Midwifery, with a degree programme in preparation [6]. However, in contrast to other ASEAN states where nursing includes midwifery, in Cambodia, midwifery and nursing have discrete pre-service education and two separate professional councils. This misalignment excludes midwifery from the MRA framework, with little obvious political leverage for change (#002).

In all health disciplines, the rise of the private sector has resulted in a surplus of graduates and uncertain standards on completion of education, with the National Exit Examination and further public service entry examinations used to control entry into the public sector. In nursing, in particular, there is an excess of graduates, but with little impact on the staffing deficits in rural Cambodia and no regional recognition of their qualifications:

Cambodia has a surplus of nurses at the moment with the proliferation of schools of nursing, and the number of graduates was huge over the last few years. I haven’t seen a massive exodus. (#002)

### Cambodian health professionals, the MRAs and mobility

Within ASEAN as a whole, there is no record of physicians or dentists relocating under the MRA provisions [3]. For nursing, mobility within ASEAN is more apparent, though no instances of Cambodian migration recorded [18], but current ASEAN health workforce migration patterns are primarily to non-ASEAN countries for all groups [19]. Interviews suggested that for doctors and medical students, institutional and personal links provided the basis for most (temporary) international placements—with *stages* in French hospitals relatively unique in offering clinical practice experience. Anecdotally, securing international qualifications enhances this possibility, as happened with JICA sponsored bridging course for nurses: “I’ve seen some invitations to me to join—to work in that hospital in Thailand” (#006). But more common is international recruitment to positions below their Cambodian status:

I heard that they are not allowed to provide direct care to the patients. They just go to assist the nurses in Thailand. (#006)

The growth of medical tourism, with private entrepreneurship in transnational provision of health care, may feed a trend towards regional mobility, recruiting Cambodians to facilitate communication with Khmer clients. In Phnom Penh, international clinics and hospitals import clinical staff—with varying degrees of compliance regarding local registration:

The new law says welcome to the real world. Everybody gets registered, everyone who needs to practice, wants to practice, has to have a license... (#002)

So every foreign nurse has to have a licence, a valid licence from the host country... the diploma, an official translation by the embassy, and also the higher level degree, but I think… nobody respects that... (#012)

We have a lot of foreign owned hospitals and health facilities and they are totally ignorant… ‘we’re better’ and therefore we don’t need to be registered in accordance with the law in this country… (#002)

But the increasing shift towards English as a primary language of instruction, economic growth and the increasing regional engagement in education are key enablers for regional and international mobility.

## Conclusions

Cambodia finds itself undergoing a series of rapid transitions: substantial demographic and economic growth since its emergence from the Khmer Rouge genocide and subsequent sanctions; increasing health status grounded in comprehensive health systems reform; and educational opportunity enabled by a shift towards increasing English usage and burgeoning private sector.

These changes have been accompanied by increasing regional engagement, though there is caution, particularly within the MoH. This is grounded in concerns around retaining its limited but hard-won local capacity, Cambodia’s vulnerability to its economically aggressive neighbours and the potential for increasing migration as standards become regionally recognised. The outcome is a tension between a conservative and hierarchical public sector and entrepreneurial innovation in the emerging but poorly regulated private sector. With health education now a shared responsibility of the MoEYS and MoH, the result is the application of a series of compensatory initiatives that seek to compensate for the irregular baseline of secondary schooling, and tertiary educational standards, and the lack of adequate enforcement of regulation. Hence, the curricula duration for medicine and dentistry is longer than regional counterparts, though it does not clearly result in equivalent outcomes. Entrance and exit examinations have been introduced because of inconsistencies in secondary schooling and unreliable institutional assessments. Nursing and midwifery education attempts to address both local need and regional demands, with limited technical or financial support. The desire to meet regional expectations in the long term is in tension with MRA guidelines in the short term: MRA acts as an international benchmark but its potential to drive internal change does not seem to have been strategically adopted. While the formal channels for regional mobility are slowly operationalised, informal processes ahead are rapidly emerging, and progress against MRA standards designed to increase inter-ASEAN mobility proceeds slowly, in contrast to poorly documented but increasing mobility beyond the ASEAN region.

## Abbreviations

ACC: Accreditation Committee of Cambodia
AEC: ASEAN Economic Community
AJCCD: ASEAN Joint Coordinating Committee Dentistry
AJCCM: ASEAN Joint Coordinating Committee Medicine
AJCCN: ASEAN Joint Coordinating Committee Nursing
ASEAN: Association of South East Asian Nations
CCN: Cambodian Council of Nurses
CMC: Cambodian Midwives Council
CPD: Continuous Professional Development
DCC: Dental Council of Cambodia
GIZ: Deutsche Gesellschaft für Internationale Zusammenarbeit
HCP: Health Coverage Plan
JICA: Japan International Cooperation Agency
KOICA: Korea International Cooperation Agency
MCC: Medical Council of Cambodia
MoEYS: Ministry of Education, Youth and Sports
MoH: Ministry of Health
MRA: Mutual Recognition Arrangements
PCC: Pharmacy Council of Cambodia
UHC: Universal Health Coverage
UHS: University of Health Sciences
WHO: World Health Organization
